# Peri-interventional Triple Therapy With Dabigatran Improves Vasomotion and Promotes Endothelialization in Porcine Coronary Stenting Model

**DOI:** 10.3389/fcvm.2021.690476

**Published:** 2021-07-02

**Authors:** Rayyan Hemetsberger, Serdar Farhan, Dominika Lukovic, Katrin Zlabinger, Judit Hajagos-Toth, Judit Bota, Hector M. Garcia-Garcia, Cihan Ay, Eslam Samaha, Robert Gaspar, Rita Garamvölgyi, Kurt Huber, Mariann Gyöngyösi, Andreas Spannbauer

**Affiliations:** ^1^Department of Cardiology and Angiology, University Hospital Bergmannsheil, Ruhr University Bochum, Bochum, Germany; ^2^Zena and Michael A. Wiener Cardiovascular Institute, Icahn School of Medicine at Mount Sinai, New York, NY, United States; ^3^Department of Cardiology, Medical University of Vienna, Vienna, Austria; ^4^Department of Pharmacology and Pharmacotherapy, University of Szeged, Szeged, Hungary; ^5^Department of Cardiology, Medstar Hospital Center, Washington, DC, United States; ^6^Clinical Division of Haematology and Haemostaseology, Department of Medicine I, Medical University of Vienna, Vienna, Austria; ^7^Institute of Diagnostics and Radiation Oncology, University of Kaposvár, Kaposvár, Hungary; ^8^3rd Medical Department of Cardiology, Wilhelminen Hospital, Vienna, Austria

**Keywords:** vasomotor function, endothelialization, neointimal formation, dabigatran, preclinical

## Abstract

**Objective:** We evaluated the short and long-term effect of peri-interventional dabigatran therapy on vasomotion, endothelialization, and neointimal formation in a porcine coronary artery stenting model.

**Background:** Stenting of coronary arteries induces local inflammation, impairs vasomotion and delays endothelialization.

**Methods:** Twenty-eight animals underwent percutaneous coronary intervention (PCI) with drug eluting stents. Sixteen pigs started dabigatran therapy 4 days prior to PCI and continued for 4 days post-stenting, while 12 animals served as controls. Post-stenting dual antiplatelet therapy (75 mg clopidogrel and 100 mg aspirin) was continued in both groups until termination. Immediately post-stenting and at day 3 optical coherence tomography (OCT) was performed in all animals, followed by euthanasia of 8 dabigatran and 4 control animals. The remaining pigs (8 of each group) were followed up for 1 month, with control angiography and OCT. Tissue burden (degree of peri-strut structure—thrombus and/or fibrin) was evaluated. After euthanasia coronary arteries were harvested for *in-vitro* myometry and histology.

**Results:** Thrombin generation was lower (*p* < 0.001) and tissue burden (0.83 ± 0.98 vs. 3.0 ± 2.45; *p* = 0.031) was significantly decreased in dabigatran treated animals. After 3 days post-PCI endothelium-dependent vasodilation was significantly improved (77 ± 40% vs. 41 ± 31%, *p* = 0.02) in dabigatran animals. Neither quantitative angiography nor histomorphometry showed differences between the groups. Endothelialization was faster in the dabigatran group as compared with controls (*p* = 0.045).

**Conclusion:** Short-term peri-interventional triple therapy with dabigatran, aspirin, and clopidogrel led to an enhanced endothelium dependent vasodilation and faster endothelialization. However, neointimal formation 1-month after stent implantation was comparable between groups.

## Introduction

Percutaneous coronary intervention (PCI) has evolved to the gold standard treatment of stenotic coronary artery disease, requiring dual antiplatelet therapy (DAPT) post-PCI (1). However, a combination of antiplatelet and anticoagulant therapy in patients with atherothrombotic risk has shown favorable ischemic outcomes compared to either antiplatelet or anticoagulant monotherapy, though with increased bleeding (2, 3).

In contrast to the intention to prevent thromboembolic events in patients requiring coronary intervention, short peri-procedural triple anticoagulant therapy might reduce the inflammatory activation with consequently decrease of neointimal hyperplasia. Thrombin is a relevant protease of the coagulation cascade and plays a central role in local vascular inflammation of coronary arteries after stenting by activating the monocyte chemoattractant factor 1 (MCP-1) and endothelial cells, and modulates vascular tone and permeability (4). The predominant mediator of thrombin signaling is the protease-activated receptor-1 (PAR-1) in most cell types, including platelets and endothelial cells (4). Therefore, our hypothesis was, that the peri-procedural triple therapy with dabigatran reduces the stenting associated inflammation by reducing thrombin and potentially modifies the coronary vasomotor function at 3 days. We were furthermore interested whether such potential early effects might have an impact on vasomotion and neointimal formation after 1 month.

Accordingly, the purpose of the present study was to evaluate the anti-inflammatory and anti-stenotic effect of a short-term peri-interventional triple therapy with dabigatran and DAPT before and after coronary stenting with drug-eluting stents (DES) in porcine coronary arteries.

## Materials and Methods

### Animal Preparation and Drug Administration

**Figure 1:**
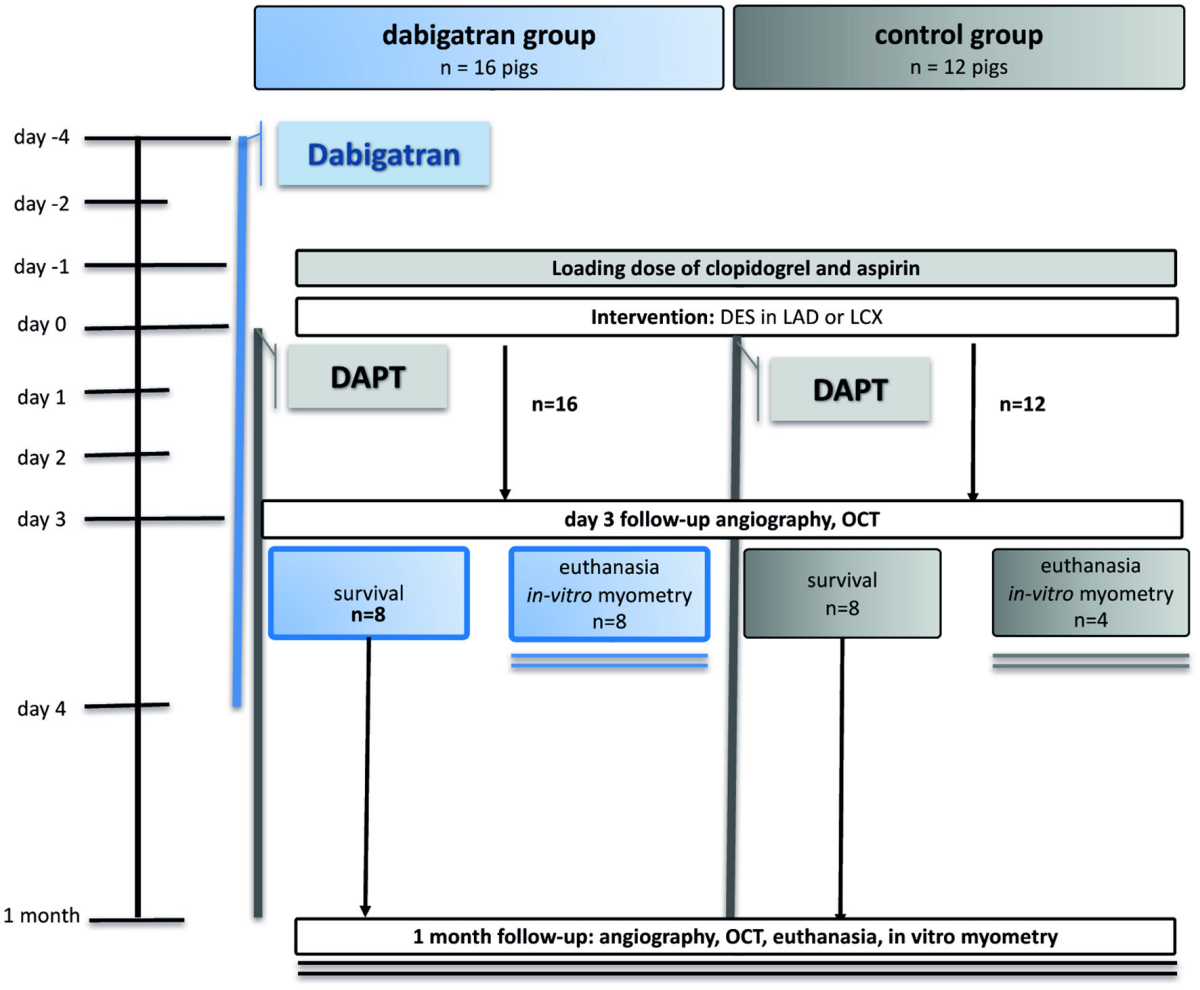
Study design. DES, drug-eluting stent; LAD, left anterior descending coronary artery; LCx, left circumflex coronary artery; DAPT, dual antiplatelet therapy; OCT, optical coherence tomography.

Twenty-eight domestic pigs (weight 32 ± 2.4 kg) were included into the study. An animal handling protocol is provided in the Supplementary Material. Four days prior to intervention sixteen of the 28 animals received 20 mg/kg dabigatran p.o. twice daily (600 mg 2x/d, equivalent to 2 × 150 mg human dose) to reach a steady state in dabigatran serum therapeutic level, according to literature data showing delayed and reduced absorption of dabigatran in pigs (5). One day before intervention all pigs (dabigatran group *n* = 16, control group *n* = 12) were loaded with 300 mg clopidogrel and 100 mg aspirin per os. All animals continued receiving 75 mg clopidogrel and 100 mg aspirin daily until euthanasia. Animals of the dabigatran group continued the anticoagulation with dabigatran up to 4 days post-stenting, since MCP-1 was described to be detected up to 4 days after intimal injury (6) (Figure 1).

At the day of PCI, anesthesia was initiated with 12 mg/kg ketamine-hydrochloride, 1 mg/kg xylazine and 0.04 mg/kg atropine and deepened with isofluorane and O_2_ via a facemask. Following intratracheal intubation, anesthesia was continued with a gas mixture of 2–3.5 vol% isofluorane, 1.6–2.5 vol% O_2_ and 0.5 vol% N_2_O. Blood pressure, O_2_ saturation and electrocardiogram were monitored. The right femoral artery was surgically prepared, an arteriotomy was performed under sterile conditions. A 7F introducer sheath (Radifocus Introducer II, Terumo Medical Corporation, Somerset, NJ, USA) was inserted into the artery. In all animals—in the control group as well as in animals treated with dabigatran—unfractionated heparin (UFH) was administered guided by activated clotting time in a range of 200–300 s.

The study was conducted in the Institute of Diagnostics and Oncoradiology, University of Kaposvar, Hungary, and approved by the Ethical Committee on Animal Experiments of the University of Kaposvar, Hungary, based on the “Principle of laboratory animal care” (NIH publication No. 86–23, revised 1985).

### Percutaneous Coronary Intervention

Guiding catheter of 7F (Medtronic, Minneapolis, MN, USA) was introduced into the left coronary artery ostium, and coronary angiography was performed using a nonionic contrast agent. After placement of a guiding wire (Cordis, Miami Lake, Florida) into the left anterior descending (LAD) and left circumflex (LCX) coronary arteries, DES (Nobori, Terumo Europe, Leuven, Belgium) (3.2 ± 0.3 mm of diameter and 20.6 ± 5.5 mm of length) were alternatingly implanted into the LAD or the LCX, according the guidelines of pre-clinical stenting experiments (7). The Nobori DES is coated only abluminally with a matrix containing Biolimus A9 and poly-lactic acid (PLA) (8).

After stenting, intravascular optical coherence tomography [OCT] imaging was performed with the C7-XR imaging system (St. Jude Medical, LightLab Imaging, Inc., Westford, Massachusetts). The image catheter (Dragonfly™, St. Jude Medical, LightLab Imaging, Inc., Westford, Massachusetts) was advanced and positioned distal to the stented segment. A continuous flush of contrast through the guiding catheter with 4 ml/s was used for blood clearance while a motorized pullback was performed. The images were captured and digitally stored by the ILUMIEN System (St. Jude Medical, St. Paul, MN) for offline analysis.

After the procedure the introducer sheath was removed, the arteriotomy was ligated, and the skin was closed in two layers. The animals were then allowed to recover from the anesthesia.

### Follow-Up

The first follow-up times was chosen 3 days after PCI since thrombus burden is highest in the first 4 days after PCI (9) and MCP-1 is upregulated in only the first 4 days after vascular injury (6, 10, 11). The final follow-up of 1-month in pigs matches a 6-month follow-up period in humans (7, 12, 13). During the follow-up period the pigs received daily 100 mg aspirin and 75 mg clopidogrel per os. The pigs of the dabigatran group received additionally dabigatran for further 4 days after intervention.

Follow-up examinations included coronary angiography, OCT followed by explanting of the heart with the stented arteries for *in vitro* myometry and histology at 3 days and 1 month. Using 10 mL of saturated potassium-chloride, 8 animals of the dabigatran group and 4 animals of the control group were sacrificed at day 3 follow-up, while 8 animals of each group were sacrificed at 1-month follow-up.

The coronary arteries were dissected, the stented segments, the proximal and distal segments adjacent to stenting were cut and flushed with physiological saline solution.

For *in-vitro* measurements of vasomotor response 4 mm long rings directly proximal and distal to the stents were cut and the measurements were performed immediately. Additionally, a 4 mm long ring from the stent free RCA was harvested, in order to have a stent free comparator.

For histopathological and histomorphometrical analysis the stented segments of the 3-day follow-up, were cut in two parts. One part was fixed in 4% formalin and was embedded in Technovit 9100, while the other part was dipped in RNAlater bath for 24 h at 4°C and then deep frozen in −20°C for quantitative real time PCR (qPCR). The 1-month follow-up stents were embedded in Technovit 9100 for histology. The plastic embedded arteries were cut into 4- to 6 μm thick slices and stained with hematoxylin-eosin.

### Analyses

All analyses were performed blinded for the treatment group.

#### Quantitative Coronary Angiography

Post-stent and follow-up quantitative coronary angiography (QCA) parameters were measured by means of a computer-assisted quantitative arteriographic edge-detection algorithm (ACOM.PC, Siemens, Erlangen, Germany).

#### OCT

Quantitative OCT measurements were performed using LightLab Imaging software (LightLab Imaging/St. Jude Medical) as previously described (14). A semi-quantitative score was developed to assess peri-strut tissue burden considered as structures on the stent strut most likely thrombus and/or fibrin (Supplementary Figure 1). First, one proximal, mid and distal section of the stent was chosen, where the tissue burden was highest. Second, the tissue burden was scored, involving the length, circumference, and thickness of peri-strut tissue. The selected cross sections were partitioned in quadrants (a, b, c, and d). One point per quadrat was given if a peri- strut tissue was detected. In case of more than 50% of the circumference within the quadrant was coated with tissue an additional point was given. As a next step the maximal peri-stent strut tissue thickness was measured for each strut in each quadrant, and additional points were given for 0.21–0.3 mm, 3 additional points for 0.31–0.5 mm, 4 additional points for 0.51–0.99 mm, and 6 additional points for ≥1mm.

#### Histomorphometry and Histopathology

The methodology for histopathology and histomorphometry is provided in the Supplementary Material.

#### Real Time Quantitative PCR (qPCR)

The stented coronary segments were analyzed using qPCR to quantify the levels of MCP-1 and PAR-1. After the stent was extracted from the artery, the tissue was transferred into RNA later (Qiagen, Germany). The non-stented RCA served as a reference control. mRNA was reverse transcribed to cDNA with QIAGEN miScript RT kit (Qiagen, Germany) and expression was quantified by qPCR with miScript SYBR® Green PCR Kit (Qiagen, Germany) on an Applied Biosystems 7500 Real-Time PCR System (Life Technologies, USA). Genes of interest were selected based on suspected involvement in in-stent restenosis development (MCP-1, PAR-1). The primers for the target sequences were designed using Primer3 software (http://primer3.wi.mit.edu/primer3web_help.htm; Microsynth, Switzerland). The method of Livak and Schmittgen (15) was used to calculate the relative expression of selected genes that were normalized to a housekeeping gene ACTB. The expression changes were calculated relative to expression levels in unstented RCA calibrator samples.

#### Thrombin Generation Assay

Thrombin generation measurements were performed directly before the intervention, immediately after stenting and at day 3 follow-up. Thrombin generation was measured using a commercially available fluorogenic assay kit (Technothrombin TGA, Technoclone, Vienna, Austria) according to manufacturer instructions (16). In this assay coagulation of platelet-poor plasma samples was initiated with addition of the TGA RC low reagent, containing a final concentration of 5 pM recombinant human tissue factor lipidated in 3.2 μmol/l phospholipid micelles (phosphatidylcholine [2.56 μmol/l] and phosphatidylserine [0.64 μmol/l]). The generated thrombin cleaves the fluorogenic substrate Z-Gly-Gly-Arg-AMC (1 mM, + 15 mM CaCl_2_) (Technoclone), while fluorescence was measured at 360 nm extinction and 460 nm emission on the same microplate reader over a period of 120 min in 1-min intervals. The peak thrombin was used in the analysis.

#### Measurements of Vasomotor Responses

Implantation of a stent, as a rigid metallic cage, results in an altered vasomotor function of the coronary arteries, adjusted to systolic-diastolic movement, and thus may result in an increased shear stress with consequent vascular complications (17). Inhibition of thrombin with consequent interruption of thrombus formation and inflammation cascade in the first days after stenting might have an influence on vasomotor function. After explanting of the heart, 4 mm long stent-free arterial ring proximal and distal adjacent to the stents were isolated, and the fat and connective tissues were removed. The segments were mounted in a temperature-controlled 15-ml tissue bath (37°C) containing a commercially available, modified Krebs–Henseleit buffer solution (K3753, Sigma-Aldrich, Vienna, Austria), as published previously (18, 19). The buffer solution was continuously controlled, and supplemented with fresh solution from the reservoir dish by using a peristaltic pump (Ismatec REGLO Digital, IDEX, Germany) to achieve a steady state flow of 1.5 ml/min. The buffer reservoir was replenished with 95% O_2_ and 5% CO_2 mixture_, to reach a pH of 7.4. To measure the isometric circular wall tension of the vessels, the segments were suspended between 2 L-shaped metal pins (0.4 mm in diameter) in a myograph (LIVING SYSTEM INSTRUMENTATION, A Division of Catamount Research and Development, Inc. Saint Albans, VT 05478, USA). After approximately 1 h stabilization period, the vessel segments were maximally contracted with endothelin-1 (30 nmol/l). Maximum endothelium-dependent vasodilation was achieved by application of substance P (1 nmol/l) directly into the organ bath. The sensitivity of smooth muscle to external nitric oxide (NO) was investigated by subsequent addition of sodium nitroprusside (4 mmol/l). The vasoconstriction, was expressed as milliNewtons (mN). The endothelium-dependent and endothelium-independent vasodilation (stiffness of vascular smooth muscle after injury of PCI) were expressed in percent change of steady-state level contraction, and in mN/s/mN units, respectively. The vasomotion experimental setup is visualized in our previous experiment (18).

#### Serological Inflammation Markers (hsCRP, IL-6, IL-1beta) and Serological Platelet Activation Marker sP-Selectin

Serum concentrations of porcine hsCRP, IL-6, IL-1beta, and sP-selectin were measured using commercial enzyme-linked immunosorbent assay (ELISA) kits. The following kits were used in this study: porcine hs-CRP (Cat.Nr.:E07C0087) (Biotrend, Cologne, Germany), porcine IL-6 (Cat.Nr.: RAB0310) (Sigma-Aldrich, St. Louis, MO, USA), swine IL-1 beta (Cat.Nr.:ESIL1B) (Thermo Fisher Scientific, Waltham, MA, USA), pig sP-selectin (Cat.Nr.: CSB-E08106p) (Cusabio, Hubei, China). All assays were performed according to the manufacturer's instructions.

### Statistics

The continuous parameters were expressed as means ± standard deviation (SD). The differences between the groups were analyzed by Student *t*-test. Statistical analyses were carried out using SPSS software (version 23.0, SPSS, IBM Corporation, NY, USA). A *p* < 0.05 was considered statistically significant.

## Results

Under the triple therapy with aspirin, clopidogrel and dabigatran (plus unfractionated heparin during the PCI procedure) one animal suffered from a hematoma at the groin at day 3 follow-up with a hemoglobin drop of 4.4 g/dl. Vital parameters were stable without tachycardia or hemodynamic compromise.

### Thrombin Assay

The anticoagulant effect of dabigatran in the pigs was measured using thrombin generation, directly before (after 4-days treatment with dabigatran in dabigatran group and in controls) and directly (30 min) after the intervention, and at day 3 follow-up. Dabigatran-treated animals had significantly lower thrombin peak levels before the stenting and at 3-days follow-up than controls (Supplementary Figure 2).

### QCA

QCA parameters at baseline, after the intervention, at day 3 follow-up and at 1 month follow-up did not differ significantly between the groups (Supplementary Table 1).

### OCT

Quantitative morphometric OCT parameters were not significantly different in the groups (Supplementary Table 2). In contrast, the tissue burden on the stent struts was significantly lower directly after the intervention in animals treated with dabigatran than in controls. This effect did not last to day 3 follow-up (Table 1).

**Table 1:** Optical coherence tomography (OCT) results of the tissue burden score immediately after coronary stenting and at day 3 follow-up.

	**Immediately after stenting**	**3 Days post-stenting**
Dabigatran Group	0.83 ± 0.98	6.87 ± 2.26
Control Group	3.0 ± 2.45	7.8 ± 3.39
*p*-value	0.031	0.42

*Means ± SD*.

### Histomorphometry and Histopathology

Similar to QCA and OCT, histomorphometrical results did not differ between the groups (Supplementary Tables 1–3).

**Figure 2:**
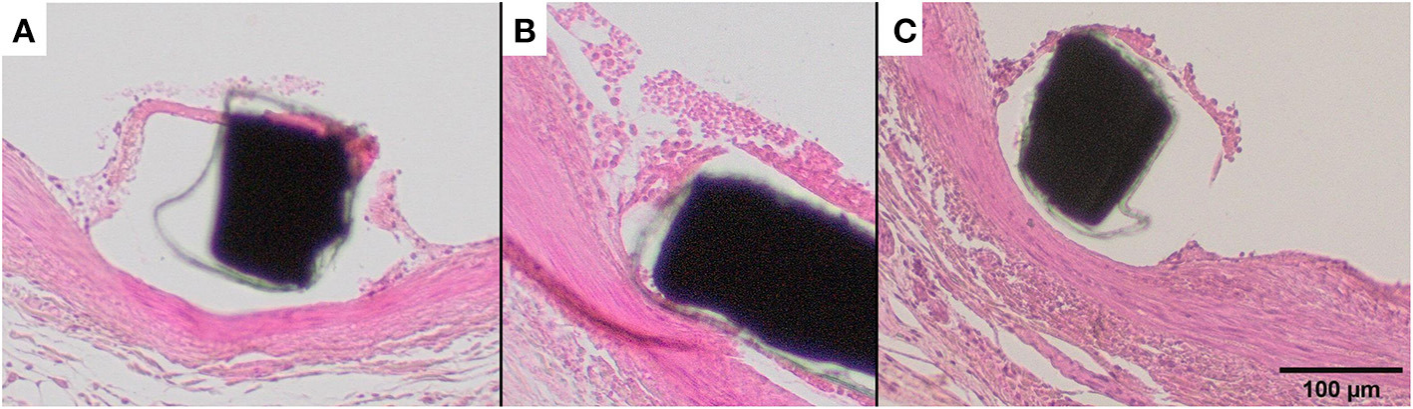
Representative histologic illustration of the tissue coverage over the stent struts at day 3 after intervention with inflammation, fibrin and endothelialization. **(A)** Tissue coverage over the stent struts with mainly fibrin, **(B)** with inflammatory cells and **(C)** with endothelial cells and leucocytes over the stent struts at day 3 after intervention, regardless of the treatment arm.

Injury score did not differ between the groups. The histopathological data did not show significant differences between the groups in inflammation or fibrin deposition at day 3 and 1-month follow-up. However, animals treated with dabigatran exhibited more endothelialization at day 3 and complete endothelialization at the 1-month follow-up, indicating a faster healing process (Table 2). As seen in Figure 2 the tissue over the stent struts at day 3 consists of endothelial cells and aggregated leucocytes.

**Table 2:** Histopathologic results.

	**Inflammation**		**Fibrin deposition**		**Endothelialization [%]**	
	**3d**	**1mo**	**3d**	**1mo**	**3d**	**1mo**
Dabigatran Group	0.44 ± 0.73	0.43 ± 0.79	2.22 ± 0.97	2.14 ± 1.57	44.44 ± 20.83	100 ± 0
Control Group	0.50 ± 0.58	0.25 ± 0.46	3.00 ± 0.82	2.38 ±1.19	18.75 ±12.50	78.13 ±33.91
*p*-value	0.90	0.60	0.19	0.75	0.045	0.11

*3d, 3-day follow-up; 1mo, 1-month follow-up, Means ± SD*.

### Vasomotor Edge Response

**Figure 3:**
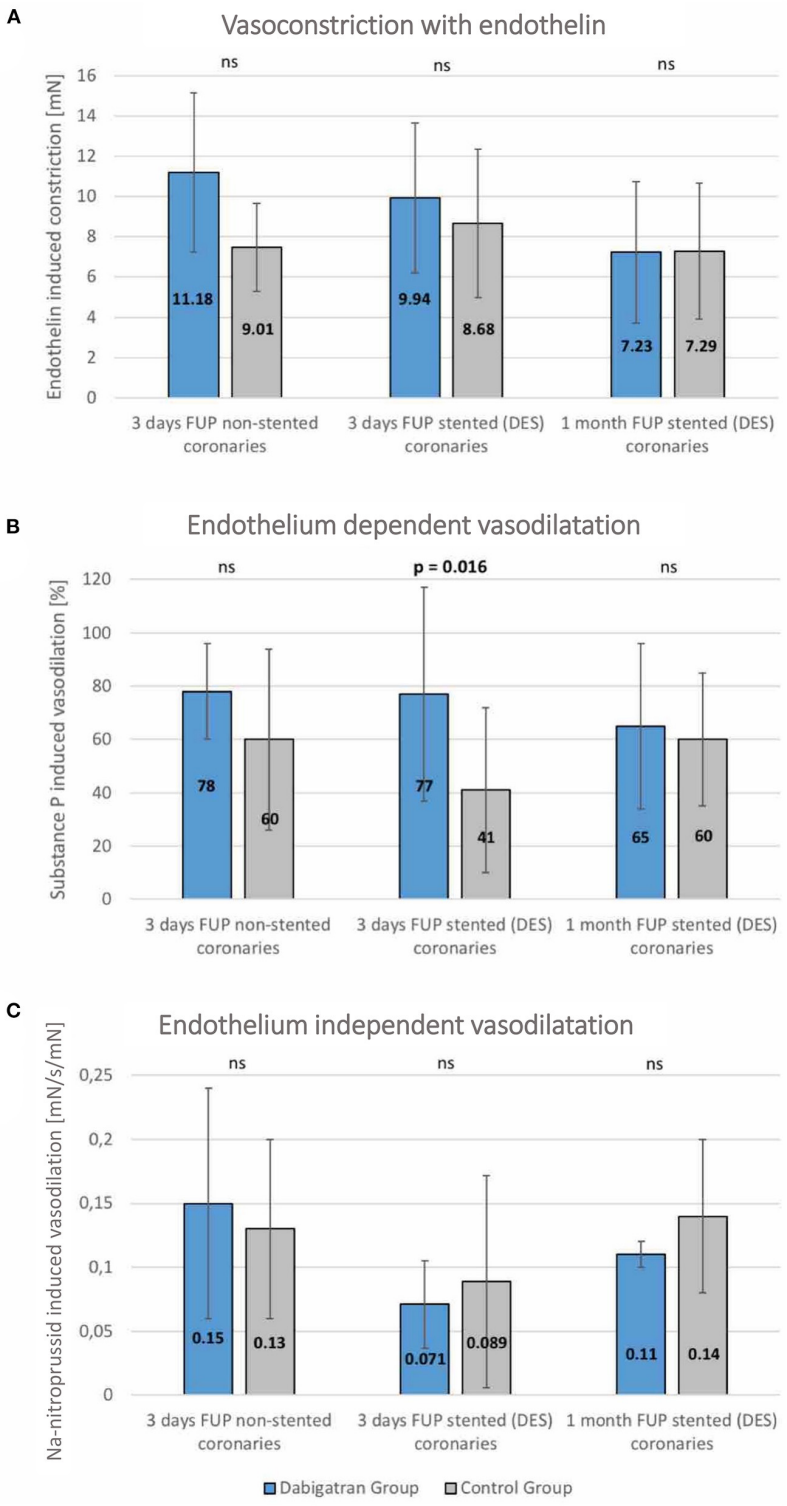
Vasomotor diagrams of stented and non-stented coronary arteries in dabigatran and control groups. **(A)** Endothelin-induced vasoconstriction, **(B)** Endothelium-dependent vasodilation, **(C)** Endothelium-independent vasodilation in non-stented (native right coronary artery) and stented (summed results of left anterior descendent/LAD/and left circumflex/LCx/) coronary arteries at 3 days post stenting, and at the 1-month follow-up. No statistically significant differences between the groups. Significantly enhanced endothelium-dependent vasodilation capacity at the 3-day follow-up in dabigatran group. Mean ± SD.

The endothelin-induced vasoconstriction did not differ between groups. At day 3 the endothelium-dependent vasodilation was significantly enhanced in the dabigatran group, whereas the endothelium independent vasodilation was similar in both treatment groups (Figure 3).

### qPCR of Tissue MCP-1 and PAR-1

**Figure 4:**
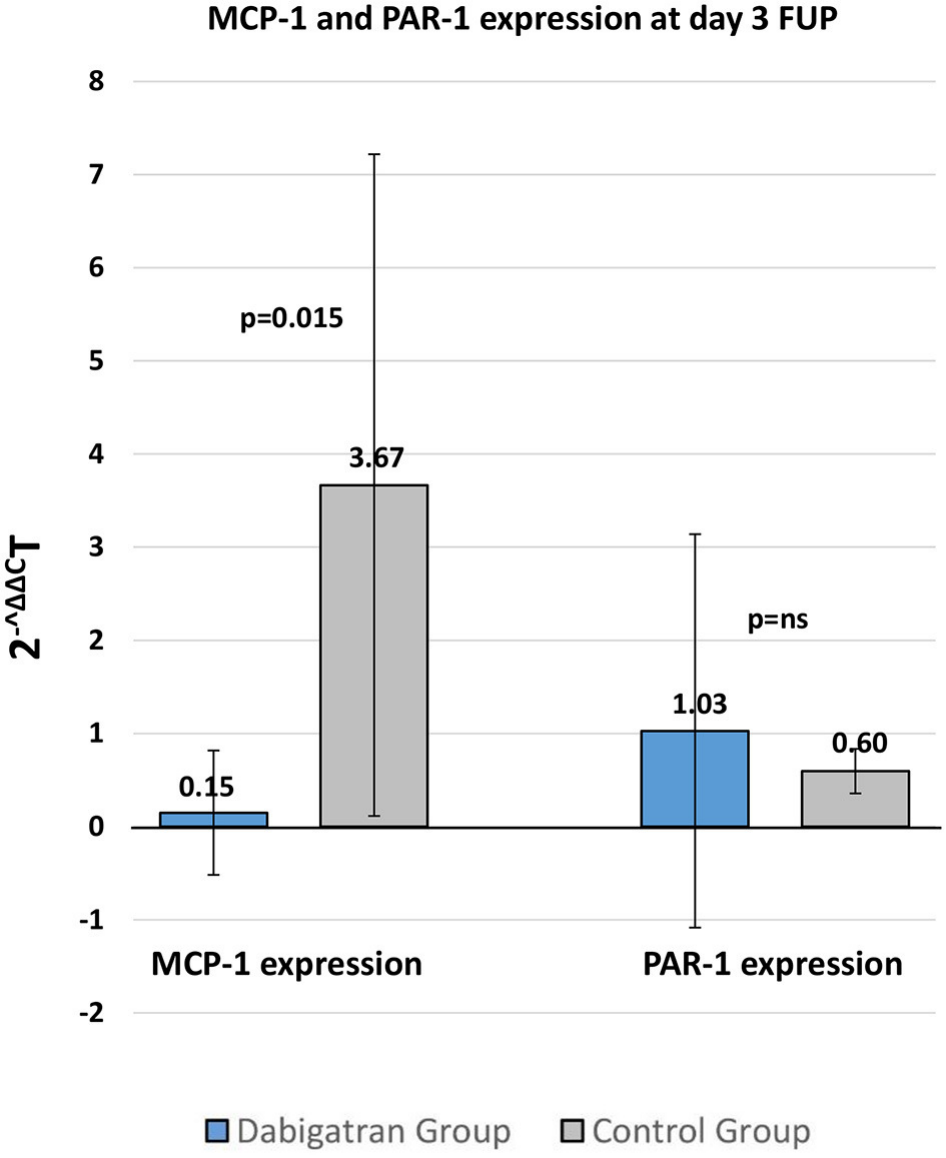
qPCR analysis of MCP-1 and PAR-1 expression of the stented tissue 3 days after stenting in dabigatran and control groups. Mean ± SD.

At day 3, during dabigatran treatment the in-stent tissue MCP1 expression was significantly lower after DES implantation. In contrast, PAR-1 expression did not show differences between the treatment groups at day 3 follow-up (Figure 4).

### Serological Inflammation Markers (hsCRP, IL-6, IL-1beta) and Serological Platelet Activation Marker sP-Selectin

During the triple therapy treatment with dabigatran, aspirin and clopidogrel hsCRP and IL-6 levels did not differ significantly as compared with the control group. However, IL-1beta levels were in the dabigatran group significantly lower at day 3 follow-up.

The serological marker for platelet activation sP-selectin had significantly lower levels in the dabigatran group as compared with controls (Supplementary Table 4).

## Discussion

In the present study investigating the impact of dabigatran on coronary artery inflammation and vessel healing after PCI with DES, we are able to report the following findings: peri-interventional treatment with dabigatran was associated with favorable effects on endothelium dependent vasodilatation, faster endothelialization as well as lower MCP-1 expression but comparable neointimal proliferation and peri-strut inflammation.

Stent implantation induces vascular injury by barotrauma associated with balloon stent inflation. Additionally foreign body reaction to the components of the stent e.g., metal and polymer are well known triggers for adverse vascular repair reaction with characteristic features such as fibrin deposition, inflammation, media necrosis and peri-strut hemorrhage (20). Dabigatran has been shown to exert antiatherosclerotic effects in mice (21). Furthermore, dabigatran treatment was associated with a dose dependent suppression of pro-inflammatory cytokines and adhesion molecules e.g., MCP-1, IL6, intercellular adhesion molecule (ICAM-1) and vascular cell adhesion molecule 1 (VCAM-1) (22). MCP-1 expressed from vascular smooth muscle cells (VSMC) and endothelial cells is known to enhance the recruitment of monocytes into the vessel wall and thereby activating an inflammatory process and subsequently increasing the risk for adverse vascular repair response to the injury induced by stent implantation (23, 24). Furthermore, MCP-1 has been shown to promote endothelial cell apoptosis (25). In the present study, peri-PCI treatment with dabigatran resulted in lower MCP-1 expression at stented segments. Taken together with a reduction of thrombin generation, the lower MCP-1 expression might explain the observed faster endothelialization in animals treated with dabigatran compared to control group with subsequently significantly higher endothelium dependent vasodilatation. In OCT we observed a lower tissue burden on the stent struts in animals treated with dabigatran directly after the intervention. However, this effect was not observed after 3 days although thrombin generation remained low. Therefore, a relationship between the lower peri-strut tissue burden initially after the intervention and the observed favorable effects on endothelium dependent vasodilatation or the faster endothelialization seems to be unlikely. Previous experimental studies however, showed that rivaroxaban, a direct inhibitor of activated factor X (FXa), enhanced endothelial growth and healing (26). Since thrombin and FXa have pleiotropic effects, as they also act via protease activated receptors, their inhibitors, both dabigatran and rivaroxaban may also have pleiotropic effects, besides to the anticoagulant action explaining our findings.

Safety concerns about dabigatran treatment arose as in the RE-LY trial a trend toward a higher incidence for myocardial infarction was observed as compared to the control group (27, 28). However, the frequency of these adverse events was low and the overall benefit and risk balance of dabigatran use appears to be favorable in patients with atrial fibrillation because of reduction in stroke. As a consequence, guidelines do not address this topic and do not recommend one specific NOAC over the other in the setting of percutaneous coronary intervention (1). Extensive research to further understand the higher incidence of myocardial infarction has shown that dabigatran enhances platelet reactivity. Dabigatran increased platelet reactivity by enhancing thrombin receptor density on platelets (29). Platelet reactivity induced by collagen, arachidonic acid and ADP was not altered by dabigatran treatment. Further an oral thrombin inhibitor treatment increased platelet adhesion and thrombus formation under flow *in vitro*. However, this prothrombotic effect was reversed by aspirin treatment (30). Taken together, the concomitant therapy of dabigatran with aspirin and clopidogrel might counteract the previously described prothrombotic effect of dabigatran. We could demonstrate that under a triple therapy with dabigatran, aspirin, and clopidogrel the serological marker for platelet activation sP-selectin was significantly lower at day 3 as compared with the control group with a treatment of aspirin and clopidogrel alone.

DES implantation in a porcine coronary artery model was associated with long-term impaired vasomotor function evidenced by a reduction in endothelium dependent vasodilatation in DES but not in bare metal stent and drug eluting balloon treated pigs (18). Similar changes were observed in an earlier experiment using first generation DES (31) although with more pronounced histological markers of inflammation. In comparison to those studies, in the present experiment, third generation DES with low strut thickness of 111 μm and biodegradable polymer was used. Such stents were shown to be associated with favorable outcomes compared to earlier DES platforms (8). This stent platform was associated with preserved endothelium-dependent vasomotion compared to first generation DES (sirolimus eluting stent) (32). Consequently, it could be hypothesized that the combined utilization of novel DES platform in combination with dabigatran resulted in the favorable findings obtained in the present study.

The findings from these pre-clinical experiments can certainly not be directly translated into clinical practice without further investigation. Although in the ATLAS ACS 2—TIMI 51 treatment with a Xa inhibitor rivaroxaban, in the lower dose of 2.5 mg, has been associated with a significant reduction of ischemic events, a higher rate of bleeding complications should be acknowledged as a major tradeoff of such treatment concept (3). In the present study under an effective anticoagulatory dose of dabigatran, and the treatment with aspirin and clopidogrel one pig suffered from access site hematoma with a hemoglobin drop of 4.4 g/dL considering it a major bleeding event. Interestingly, an uninterrupted oral anticoagulation therapy in patients undergoing unplanned percutaneous coronary intervention was associated with shorter length of hospitalization and equivalent risk for MACCE and bleeding complications as compared to interrupted oral anticoagulation therapy (33). Taking this data in consideration, the additional information on faster endothelialization and enhanced endothelium-dependent vasodilation is of interest.

## Limitations

As an experimental study the presented data should be interpreted with caution and considered hypothesis generating. The experiment was conducted on young healthy porcine coronary arteries, not representative for atherosclerotic human coronary arteries. It should be noted that the coronaries of juvenile pigs endothelialize more rapidly following injury. However, being similar to humans, the anatomy and pathophysiology of porcine coronary arteries make this PCI-model well accepted and established for basic and pre-clinical translational research (7). OCT assessment of peri-strut tissue burden was semiquantitative. Endothelialization was assessed by light microscopy and not by scanning electron microscopy. For the vasomotor responses we used 4 mm vessel rings, however we did not normalize the contraction with the dry weight of the vessel ring. Finally, in the present study, we utilized a third generation thin strut DES with biodegradable polymer (8). Such stents were associated with low restenosis and thrombosis rates as compared to early generation DES (8). This might have contributed to the different results obtained in our study compared with previous investigations (18, 31).

## Conclusion

Short-term peri-interventional triple therapy with dabigatran, aspirin, and clopidogrel led to an enhanced endothelium dependent vasodilation and faster endothelialization, however, not reducing neointimal formation 1-month after stent implantation, even if endothelialization was complete.

## Data Availability Statement

The datasets presented in this article are not readily available as the data is property of the study sponsor. Requests to access the datasets should be directed to rayyan.hemetsberger@hotmail.com.

## Ethics Statement

The animal study was reviewed and approved by Ethical Committee on Animal Experiments of the University of Kaposvár, Hungary.

## Author Contributions

RH: conceptualization, methodology, validation, formal analysis, investigation, resources, writing the original draft, visualization, project administration, and funding acquisition. SF: conceptualization, methodology, writing-review and editing, and visualization. DL, KZ, JH-T, JB, CA, ES, RGas, and RGar: validation, formal analysis, investigation, resources, and writing-review and editing. HG-G: methodology, writing-review and editing, and visualization. KH: validation, formal analysis, investigation, resources, writing-review and editing, and supervision. MG: conceptualization, methodology, validation, formal analysis, investigation, resources, writing-review and editing, visualization, project administration, funding acquisition, and supervision. AS: methodology, validation, formal analysis, investigation, resources, writing-review and editing, and visualization. All authors contributed to the article and approved the submitted version.

## Conflict of Interest

KH reports lecture and consultation fees from Astra Zeneca, Bayer, Boehringer Ingelheim, Bristol Myers Squibb, Daiichi Sankyo Pfizer, and Sanofi with this manuscript. The remaining authors declare that the research was conducted in the absence of any commercial or financial relationships that could be construed as a potential conflict of interest.

## Nomenclature

DES, drug-eluting stent(s)

DAPT, dual antiplatelet therapy

LAD, left anterior descending coronary artery

LCX, left circumflex coronary artery

MCP-1, monocyte chemoattractant protein 1

NOAC, non vitamin K antagonist oral anticoagulant

OCT, optical coherence tomography

PCI, percutaneous coronary intervention

PAR-1, protease activated receptor 1

QCA, quantitative coronary angiography

VSMC, vascular smooth muscle cells.
