# Supplementary material for: Peri-interventional Triple Therapy With Dabigatran Improves Vasomotion and Promotes Endothelialization in Porcine Coronary Stenting Model
Source: Front Cardiovasc Med. 2021 Jul 2;8:690476. doi: 10.3389/fcvm.2021.690476 (PMC8300015; doi:10.3389/fcvm.2021.690476)
Supplement: Supplementary file 3 [file Data_Sheet_1.docx]

**Supplemental Material**

**Methods and Material**

### *Animal handling protocol*

The pigs are housed under controlled and standardized conditions (artificial day-night rhythm 12:12; room temperature 22±2°C. humidity 55±10%) in groups. the enclosures are outfitted with straw on the floors. After arrival at the facilities. the pigs are acclimated to their new environment for 1 week before experiments. The pigs are fed with a special feed for pigs (LASvendi. Germany). apples as needed and water ad libitum. The animals are checked twice daily by animal handlers.

Indications for premature termination of experiments and euthanasia were:

- rapid decrease in body weight up to 20%
- Refuse of food- and/or water uptake
- uncontrolled bleeding
- uncontrolled infection
- observed behavior as a sign for significant pain
- contamination of surgical site
- respiratory disorders
- significant change in behavior or neurological disabilities

### *Histomorphometry and Histopathology*

Histopathology and histomorphometry assessments were carried out by experienced observers without knowledge of the stent type or medication group. Each stented segment was cut into 3 parts (proximal. middle. and distal) and were evaluated using the recommendation of Schwartz et al. (1).

Briefly. inflammation score was graded as 0 for no inflammation or minimal amount of inflammatory cells in media and adventitia; 1 for mild diffuse inflammatory infiltration or focally moderated in <25% of the vessel area in media and adventitia; 2 for moderate inflammatory infiltration or focally marked in 25-50% of the vessel area in media and adventitia; 3 for heavy inflammatory infiltration or focally marked in >50% of the vessel area in media and adventitia. and 4 for granulomatous inflammatory reaction in any vessel wall layer of the artery. Fibrin score was graded from 0 to 3 as no fibrin deposition or fibrin deposition involving <10%. 10-25% or >25% of the circumference of the vessel. respectively. The severity of injury was graded: Grade 0 (no injury): internal (IEL) and external elastic lamina (IEL) and media intact; Grade 0.5: IEL minimal disruption. media and EEL intact; Grade 1: IEL lacerated. media and EEL intact; Grade 1.5 IEL lacerated. media <half thickness lacerated. EEL intact; Grade 2: IEL lacerated. media >half thickness lacerated. EEL intact; Grade 2.5: IEL and media (full thickness) lacerated. IEL minimal disruption; Grade 3: IEL. media (full thickness) and EEL lacerated.

Endothelial coverage was expressed as the percentage of the lumen circumference covered by endothelium. measured by computerized planimetry (ImageJ version 1.440. NIH. Bethesda. Maryland).

The following quantitative histomorphometric parameters were measured: lumen. internal and external elastic lamina area. and the maximal neointimal thickness. The calculated histomorphometric parameters included the neointima area (difference between internal elastic lamina and lumen area). media area (difference between external and internal elastic lamina area). and % area stenosis [(neointimal area/internal elastic lamina area) x 100].

A matched analysis of in vivo OCT and ex vivo histology could not be performed. since no external marker could be implanted to serve as a reference to ensure the precise correlation of individual cross sections.

**Supplementary Figure Legends**

**Supplementary figure 1. Representative illustration for assessing tissue burden score in optical coherence tomography image.**

The semi-quantitative score of tissue burden (consisting of thrombus and inflammatory cells 3-days post stenting) assessed the length. circumference and maximal thickness of the peri-strut tissue. Briefly. stent cross-sections of the proximal. mid and distal stent with the most tissue burden were selected and divided into 4 quadrants. Summed score was calculated for each stent by considering the presence. extent. and maximal thickness of peri-stent strut structure.

**Supplementary figure 2. Thrombin peak levels.**

Thrombin generation directly before and after the intervention. and at day 3 follow-up in dabigatran and control groups. Pigs in dabigatran group received dabigatran 4 days before and 4 days after coronary stenting. Directly after the intervention due to heparin administration no thrombin peak was measurable in both groups. Mean±SD.

**Supplementary Tables**

**Supplement Table S1. Quantitative angiographic results immediately after coronary stenting (percutaneous coronary intervention /PCI/. at day 3 and at 1-month follow-up (FUP).**

post-PCI: immediately after PCI. RD: reference diameter. MLD: minimal lumen diameter. %DS: percent diameter stenosis. LLL: late lumen loss. means±SD.

1. Baseline and 3-day FUP results

|  | **baseline**  **RD**  (mm) | **post-PCI**  **MLD**  (mm) | **post-PCI**  **%DS**  (%) | **3d FUP**  **RD**  (mm) | **3d FUP**  **MLD**  (mm) | **3d FUP**  **%DS**  (%) | **3d FUP**  **LLL**  (mm) |
| --- | --- | --- | --- | --- | --- | --- | --- |
|  |  |  |  |  |  |  |  |
| **dabigatran** | **2.81** | **3.03** | **3.24** | **3.23** | **3.03** | **5.92** | **0.00** |
| **group** | ±0.59 | ±0.45 | ±5.53 | ±0.28 | ±0.29 | ±5.39 | ±0.38 |
|  |  |  |  |  |  |  |  |
| **control** | **2.72** | **3.00** | **1.08** | **3.22** | **2.96** | **8.22** | **0.01** |
| **group** | ±0.4 | ±0.26 | ±2.15 | ±0.32 | ±0.32 | ±4.55 | ±0.26 |
|  |  |  |  |  |  |  |  |
| **p value** | 0.62 | 0.84 | 0.21 | 0.99 | 0.54 | 0.27 | 0.92 |
|  |  |  |  |  |  |  |  |

1. One-month FUP results

|  | **1mo FUP**  **RD**  (mm) | **1mo FUP**  **MLD**  (mm) | **1mo FUP**  **%DS**  (%) | **1mo FUP**  **LLL**  (mm) |
| --- | --- | --- | --- | --- |
|  |  |  |  |  |
| **dabigatran** | **3.15** | **2.65** | **16.36** | **0.33** |
| **group** | ±0.37 | ±0.75 | ±20.10 | ±0.62 |
|  |  |  |  |  |
| **control** | **3.07** | **2.37** | **23.38** | **0.67** |
| **group** | ±0.49 | ±0.80 | ±20.14 | ±0.80 |
|  |  |  |  |  |
| **p value** | 0.70 | 0.48 | 0.50 | 0.36 |
|  |  |  |  |  |

**Supplement Table S2. Quantitative optical coherence tomographic results immediately after coronary stenting (percutaneous coronary intervention /PCI/ at day 3 and at 1-month follow-up (FUP).**

post-PCI: immediately after PCI. means±SD.

|  | **post-PCI**  **lumen area**  (mm^2^) | **3-day FUP**  **lumen area**  (mm^2^) | **1-month FUP**  **lumen area**  (mm^2^) | **1-month FUP**  **area stenosis**  (%) |
| --- | --- | --- | --- | --- |
|  |  |  |  |  |
| **dabigatran** | **8.18** | **7.99** | **5.25** | **25.47** |
| **group** | ±1.14 | ±0.95 | ±1.45 | ±18.53 |
|  |  |  |  |  |
| **control** | **8.42** | **8.51** | **5.65** | **26.12** |
| **group** | ±0.98 | ±1.31 | ±2.28 | ±21.68 |
|  |  |  |  |  |
| **p value** | 0.67 | 0.28 | 0.69 | 0.95 |
|  |  |  |  |  |

**Supplement Table S3. Histomorphometric results.**

3d: 3-day follow-up. 1mo: 1-month follow-up. means±SD.

|  |  |  |  |  | **Max intimal thickness**  (mm) | |  |  |  |  |  |  |  |  |
| --- | --- | --- | --- | --- | --- | --- | --- | --- | --- | --- | --- | --- | --- | --- |
|  | **lumen area**  (mm^2^) | | **intimal area**  (mm^2^) | |  |  | **IEL area**  (mm^2^) | | **EEL area**  (mm^2^) | | **medial area**  (mm²) | | **stent stenosis**  (%) | |
|  | **3d** | **1mo** | **3d** | **1mo** | **3d** | **1mo** | **3d** | **1mo** | **3d** | **1mo** | **3d** | **1mo** | **3d** | **1mo** |
|  |  |  |  |  |  |  |  |  |  |  |  |  |  |  |
| **dabigatran** | **5.22** | **4.11** | **0.34** | **1.64** | **0.04** | **0.31** | **5.55** | **5.75** | **6.49** | **7.54** | **0.94** | **0.73** | **6** | **27.9** |
| **group** | ±1.72 | ±0.39 | ±0.25 | ±0.60 | 0.02 | 0.17 | ±1.91 | ±0.82 | ±2.00 | ±1.46 | 0.30 | 0.50 | ±3 | ±7 |
|  |  |  |  |  |  |  |  |  |  |  |  |  |  |  |
| **control** | **4.69** | **2.82** | **0.19** | **1.74** | **0.04** | **0.22** | **4.87** | **5.56** | **5.86** | **7.37** | **0.98** | **0.83** | **4** | **31.7** |
| **group** | ±0.70 | ±1.21 | ±0.13 | ±0.8 | 0.02 | 0.15 | ±0.61 | ±1.09 | ±0.49 | ±1.01 | 0.22 | 0.36 | ±3 | ±16 |
|  |  |  |  |  |  |  |  |  |  |  |  |  |  |  |
| **p value** | 0.57 | 0.59 | 0.30 | 0.83 | 0.92 | 0.32 | 0.51 | 0.74 | 0.55 | 0.82 | 0.81 | 0.67 | 0.37 | 0.62 |
|  |  |  |  |  |  |  |  |  |  |  |  |  |  |  |

**Supplement Table S4. Serological inflammatory markers hsCRP. IL-6. IL-1-beta and serological platelet activation marker sP-selectin.**

Baseline: before dabigatran intake/at baseline; 3d: 3-day follow-up; means±SD.

|  |  |  |  |  |  |  | |  | |  |
| --- | --- | --- | --- | --- | --- | --- | --- | --- | --- | --- |
|  | **hsCRP** [ng/ml] | | **IL-6** [pg/ml] | | **IL-1beta** [pg/ml] | | | **sP-selectin** [ng/ml] | | |
|  | **baseline** | **3d** | **baseline** | **3d** | **baseline** | | **3d** | **baseline** | **3d** | |
|  |  |  |  |  |  | |  |  |  | |
| **dabigatran** | **42.9** | **31.3** | **39.1** | **68.4** | **524.8** | | **377.81** | **0.72** | **0.0** | |
| **group** | ±12.0 | ±17.5 | ±101.5 | ±94.7 | ±866.1 | | ±843.41 | ±1.61 | ±0.0 | |
|  |  |  |  |  |  | |  |  |  | |
| **control** | **39.7** | **30.2** | **74.4** | **150.6** | **1499.6** | | **1551.2** | **3.40** | **27.21** | |
| **group** | ±12.8 | ±12.0 | ±105.8 | ±259.2 | ±1864.2 | | ±1731.3 | ±7.60 | ±49.20 | |
|  |  |  |  |  |  | |  |  |  | |
| **p value** | 0.53 | 0.87 | 0.43 | 0.38 | 0.32 | | **0.02** | 0.46 | **0.03** | |

**References**

1. Schwartz RS. Edelman E. Virmani R et al. Drug-eluting stents in preclinical studies: updated consensus recommendations for preclinical evaluation. Circ Cardiovasc Interv 2008;1:143-53.
